# Cannabis Use and Diet Quality Among University Students: The Role of Meal Skipping and Health Behaviours

**DOI:** 10.3390/nu18132210

**Published:** 2026-07-07

**Authors:** Rawan Alfares, Jasna Twynstra, Jason A. Gilliland, Jamie A. Seabrook

**Affiliations:** 1 Brescia School of Food and Nutritional Sciences, Western University, London, ON N6G 2V4, Canada; ralfare@uwo.ca (R.A.); jjunuzo@uwo.ca (J.T.); 2 Children’s Health Research Institute, London, ON N6C 2V5, Canada; jgillila@uwo.ca; 3 Human Environments Analysis Laboratory, Western University, London, ON N6A 3K7, Canada; 4 Department of Geography and Environment, Western University, London, ON N6A 5C2, Canada; 5 Department of Epidemiology and Biostatistics, Western University, London, ON N6G 2M1, Canada; 6 Department of Paediatrics, Western University, London, ON N6A 5W9, Canada; 7 Lawson Research Institute, London, ON N6A 4V2, Canada; 8 London Health Sciences Centre Research Institute, London, ON N6A 5W9, Canada; 9 School of Health Studies, Western University, London, ON N6A 3K7, Canada

**Keywords:** cannabis use, diet quality, university students, meal skipping, behavioural clustering, young adults, nutrition, health behaviours, Canadian Food Intake Screener, substance use

## Abstract

**Background/Objectives:** Diet quality among university students is influenced by multiple behavioural and lifestyle factors, yet limited research has examined how cannabis use relates to overall diet quality within this population. This study examined the association between cannabis use and diet quality among university students and assessed whether this relationship was explained by behavioural, contextual, and psychological factors. **Methods:** A cross-sectional online survey was distributed to all registered students at a large Canadian university in January 2026. Diet quality was assessed using the Canadian Food Intake Screener (CFIS), and past 30-day cannabis use was examined as the primary exposure. Hierarchical multiple linear regression models were conducted sequentially, adjusting for demographic characteristics, health behaviours, mental health variables, living arrangements, meal skipping, and other substance use. **Results:** Among 1581 survey respondents, 1467 participants were included in the fully adjusted regression analyses. Past 30-day cannabis use was reported by 33.7% of participants. In demographic-adjusted analyses, cannabis use was associated with lower diet quality scores (B = −0.81, *p* < 0.01). This association remained statistically significant following adjustment for health behaviours, mental health variables, and living arrangements. However, after adjustment for meal skipping, the association between cannabis use and diet quality was attenuated and no longer statistically significant (B = −0.44, *p* = 0.09). Meal skipping emerged as one of the strongest behavioural correlates of lower diet quality. Additional adjustment for other substance use did not materially alter findings. **Conclusions:** Cannabis use was initially associated with lower diet quality among university students; however, this association was attenuated after accounting for broader behavioural factors, particularly meal skipping. Given the cross-sectional design, these findings do not establish whether cannabis use influences dietary behaviours or whether meal skipping represents a pathway linking cannabis use and diet quality. These findings highlight the importance of considering diet quality within a broader behavioural framework and suggest that eating patterns represent an important correlate of diet quality among university students.

## 1. Introduction

Diet quality is an important determinant of long-term physical and mental health, with poor dietary patterns linked to increased risk of obesity, cardiometabolic disease, and poorer cognitive and academic performance [1]. Despite its importance, diets that do not meet dietary recommendations remain highly prevalent among young adults, particularly those enrolled in postsecondary education [2]. Young adulthood represents a critical developmental stage for health, during which individuals experience increased independence alongside transitions in living arrangements, food environments, and broader life responsibilities [3,4]. Within this life stage, university students represent an important subgroup of young adults who are often navigating these transitions while pursuing postsecondary education. Dietary behaviours in this population are characterized by a high prevalence of meal skipping and frequent consumption of energy-dense, nutrient-poor foods, alongside insufficient intakes of fruits, vegetables, whole grains, and legumes [2]. However, research examining overall diet quality among university students within a broader behavioural context remains limited. Most existing studies focus on individual dietary behaviours rather than considering diet in relation to co-occurring lifestyle factors, despite evidence that health behaviours tend to cluster within individuals [5]. This highlights the importance of examining diet quality within an integrated behavioural framework rather than in isolation. Despite growing interest in health behavioural clustering among young adults, little is known about how cannabis use relates to overall diet quality within university student populations.

Among the range of lifestyle factors that may influence dietary behaviours in young adults, substance use behaviours represent a particularly relevant area of interest. In this context, cannabis use is one of the most commonly used substances among university students in Canada [6]. In a cross-sectional survey of 744 undergraduate students at Carleton University, 52.4% reported past-month cannabis use [6]. Among cannabis users, 44.8% were classified as having hazardous cannabis use based on the Cannabis Use Disorder Identification Test–Revised (CUDIT-R), a validated screening tool used to identify patterns of cannabis consumption associated with increased risk of harm or cannabis use disorder [6]. Another cross-sectional study of 2626 students from Western University found that 36.7% of 17–19-year-olds, 41.1% of 20–25-year-olds, and 35.6% of students aged 26+ years had used cannabis in the past month [4]. Given the high prevalence of cannabis use within student populations, there is growing interest in its potential associations with health-related behaviours, including dietary intake and eating patterns. Cannabis has been shown to influence appetite regulation and acute food intake, with evidence consistently demonstrating increased energy intake following use, often characterized by increased cravings for highly palatable foods [7]. Although cannabis use has been consistently associated with increased appetite and caloric intake, these acute effects do not necessarily translate into differences in long-term dietary quality. For example, using NHANES data, Gelfand et al. reported that current cannabis users had lower Healthy Eating Index (HEI) scores compared with former and never users, with differences observed across several dietary components, including fruits, vegetables, greens and beans, and whole grain intake [8]. However, findings regarding cannabis use and broader dietary patterns remain limited, particularly among younger populations and university students. Moreover, cannabis use among university students also commonly occurs alongside other substance use behaviours, reflecting broader patterns of polysubstance use in this population [9].

The relationship between cannabis use and diet quality may not be straightforward, as dietary behaviours may be influenced by broader lifestyle patterns. For example, lower physical activity, shorter sleep duration, higher sedentary behaviour, and substance use commonly co-occur, and each has been associated with poorer diet quality [10]. Similarly, poorer mental health, including higher stress and anxiety, has also been associated with lower diet quality among university students [11]. Certain dietary behaviours that are common in student populations may also overlap with cannabis use. In a meta-analysis of 71 studies assessing consumption patterns of energy drinks in undergraduate university students, the estimated overall prevalence of energy drink consumption was 42.9% and was frequently correlated with alcohol use and smoking [12]. Within university student populations, irregular eating behaviours, such as meal skipping, are also highly prevalent and associated with lower overall diet quality [10]. Additionally, students’ living situations may be associated with diet quality. Evidence suggests, for example, that those living away from home often have lower dietary quality, largely due to economic constraints and challenges in maintaining regular meal routines [13]. Within this framework, cannabis use may reflect broader behavioural patterns associated with poorer diet quality. However, the extent to which cannabis-diet associations are explained by co-occurring health behaviours remains unclear.

Therefore, this study examined the association between cannabis use and diet quality among university students. In addition to demographic characteristics, analyses considered behavioural, contextual, and psychological factors known to be associated with dietary behaviours, including physical activity, sedentary behaviour, sleep duration, meal skipping, living arrangements, energy drink consumption, and symptoms of anxiety and depression. Because cannabis use often co-occurs with other substance use behaviours in young adults, analyses also accounted for polysubstance use. We hypothesized that cannabis use would be associated with poorer diet quality after adjustment for demographic, behavioural, contextual, and psychological factors.

## 2. Materials and Methods

### 2.1. Study Design and Participants

A cross-sectional online survey was administered using Qualtrics and distributed by the Office of the Registrar to all registered students at Western University in January 2026 via mass email. All registered students were eligible to participate. Two reminder emails were sent at one-week intervals. The survey assessed cannabis use, other substance use, nutrition-related behaviours, mental health, sleep, physical activity, and selected demographic characteristics. The first page of the survey contained the Letter of Information, which outlined the study purpose, procedures, risks and benefits, confidentiality, and participant rights. Participants who proceeded to the survey after reviewing the Letter of Information provided implied consent to participate. Participation was voluntary and anonymous. Upon survey completion, participants could enter a draw for one of ten CAD $50 Amazon gift cards. A total of 38,360 students were invited to participate, of whom 1581 completed the survey and were included in the analytic sample, representing a response rate of a 4.1%.

The survey was pilot tested with five individuals external to the university and research team to assess clarity and survey flow. No changes were required based on pilot feedback. Completion time was approximately 15 to 20 min.

The study received approval from the Non-Medical Research Ethics Board at Western University (127886).

#### 2.1.1. Outcome: Diet Quality

The primary outcome was overall diet quality, measured using the Canadian Food Intake Screener (CFIS). The CFIS is a validated 16-item instrument that assesses alignment with the healthy eating recommendations in Canada’s Food Guide [14]. Although validation studies have not specifically focused on university undergraduate students, the instrument was developed for use among Canadian populations and is appropriate for assessing diet quality in large-scale survey research settings. Participants reported the frequency with which they consumed various food and beverage groups over the previous month using standardized response options. Responses were scored according to established CFIS procedures to generate a continuous total score ranging from 0 to 65, with higher scores indicating greater alignment with Canada’s Food Guide and overall diet quality [14].

#### 2.1.2. Exposure: Cannabis Use

The primary exposure was recent cannabis use. Participants reported both lifetime cannabis use and past 30-day cannabis use. For the present analyses, past 30-day cannabis use was selected a priori as the primary exposure indicator and dichotomized as yes/no to distinguish recent cannabis users from non-users because several frequency categories contained small cell sizes that reduced model stability.

#### 2.1.3. Demographic Covariates

Demographic covariates included age, gender, and race/ethnicity. Age was analyzed as a continuous variable in years. Gender was measured using categorical response options, including male, female, self-identified gender, and prefer not to answer. For analysis, gender was restricted to male and female due to small sample sizes in other categories, which limited statistical stability. Participants self-identified race/ethnicity from multiple categories: White, South Asian, Chinese, Black, Filipino, Arab, Latin American, Southeast Asian, West Asian, Korean, Japanese, Indigenous, or Other. For regression analyses, categories were collapsed into White, South Asian, Chinese, and Other Ethnicity to improve statistical stability and cell sizes, particularly given the relatively small number of participants within several individual racial/ethnic categories. This approach was intended to reduce sparse data bias within regression models; however, it may mask meaningful heterogeneity among diverse populations grouped within the Other Ethnicity category. Findings related to race/ethnicity should therefore be interpreted cautiously and should not be considered representative of individual populations included within aggregated categories.

#### 2.1.4. Health Behaviour Covariates

Health behaviours included moderate-to-vigorous physical activity (MVPA), sedentary behaviour, sleep duration, and energy drink consumption. MVPA was assessed as hours per week engaged in MVPA, with response options ranging from 0 h to 8 or more hours per week. Sedentary behaviour was assessed as hours per day spent in recreational sedentary activities (e.g., television viewing, phone use, gaming), with response options ranging from 0 h to 8 or more hours per day. Sleep was assessed using bedtime and wake time categories; responses were converted to approximate numeric midpoints to estimate average nightly sleep duration. Energy drink consumption was assessed as frequency of intake during the previous seven days, with response options ranging from “never” to “two or more times per day”.

#### 2.1.5. Mental Health Covariates

Mental health was assessed using two validated screening tools: the Patient Health Questionnaire-2 (PHQ-2) to measure depressive symptoms [15] and the Generalized Anxiety Disorder-2 (GAD-2) to measure anxiety symptoms [16]. Participants reported symptom frequency over the previous two weeks using four-point response scales ranging from “not at all” to “nearly every day.” Responses were summed to generate continuous scores ranging from 0 to 6, with higher scores indicating greater symptom severity.

#### 2.1.6. Contextual Covariate

Contextual covariates included current living arrangement, categorized as campus residence, off-campus alone, off-campus with roommates, off-campus with parents, and off-campus with a common law partner/spouse.

#### 2.1.7. Sensitivity Variables

Meal skipping was included as a sensitivity variable because it conceptually overlaps with overall diet quality and may represent either a behavioural correlate or a potential pathway linking cannabis use with dietary behaviours. Because adjustment for variables occurring on the causal pathway may introduce overadjustment bias and attenuate observed associations, findings from this model were interpreted cautiously and considered exploratory rather than confirmatory. Participants reported the number of days during the previous seven days they skipped breakfast, lunch, and dinner, with response options ranging from 0 to 7 days for each meal. Responses were summed to create a continuous meal skipping score, with higher scores indicating more frequent meal skipping.

Additional sensitivity analyses adjusted for other substance use. Participants reported past 30-day use of alcohol, cigarettes, vaping products, cocaine, psychedelics, and non-prescribed stimulants. These variables were combined into a binary indicator of any other substance use (yes/no).

### 2.2. Statistical Analysis

All analyses were conducted using IBM SPSS Statistics for Windows, Version 30.0 (IBM Corp., Armonk, NY, USA). The mean and standard deviation (SD) were used to summarize continuous variables, whereas percentages were used for categorical variables. Pearson correlation coefficients were used to examine associations between diet quality and study covariates prior to multivariable modelling. Correlation coefficients were interpreted using Cohen’s conventional effect size guidelines, whereby absolute correlation coefficients (|r|) of approximately 0.10, 0.30, and 0.50 represent small, medium, and large associations, respectively [17]. Hierarchical multiple linear regression models were used to examine the association between cannabis use and CFIS diet quality scores. Model 1 included cannabis use and demographic covariates. Model 2 additionally adjusted for health behaviours (i.e., energy drink consumption, sedentary behaviour, MVPA, and sleep duration). Model 3 further adjusted for mental health variables (i.e., PHQ-2, GAD-2). Model 4 additionally included the living arrangement.

Two sensitivity analyses were conducted. Model 5 was adjusted for meal skipping, which was included as an exploratory sensitivity analysis because meal skipping may conceptually overlap with diet quality and could also represent a behavioural pathway linking cannabis use with diet quality. Accordingly, the findings from Model 5 were interpreted cautiously. Model 6 further adjusted for any other substance use to examine whether associations were independent of broader substance use patterns.

Collinearity diagnostics were assessed using variance inflation factors (VIF), which ranged from 1.01 to 1.89, indicating no evidence of problematic multicollinearity. Regression assumptions were evaluated through inspection of residual plots for linearity, normality, and homoscedasticity diagnostics. Analyses were conducted using complete cases for variables included in each model. The proportion of missing data was low across included variables. Participants were included in the analytic sample if they provided sufficient information to calculate the CFIS diet quality score and reported their past 30-day cannabis use. Participants with missing data for individual covariates were excluded only from regression models that included those covariates, consistent with complete-case analysis. Survey responses were reviewed for duplicate entries, and no duplicate responses were identified. Statistical significance was set at *p* < 0.05.

A complete description of survey variables and coding is provided in Supplementary Table S1.

## 3. Results

### 3.1. Sample Characteristics

Of the 1581 participants who completed the survey, analytic sample sizes ranged from 1479 in the baseline model to 1467 in the fully adjusted model due to missing data on specific covariates. The mean age of participants was 21.6 years (SD = 5.2), and 74.4% of the sample were female. Overall, 33.7% of participants reported cannabis use in the past 30 days. Over half (53.0%) of the participants identified as White, 14.7% as South Asian, 12.1% as Chinese, and 21.2% as Other Ethnicity.

#### Health Behaviours and Other Characteristics

Participants reported a mean of 2.9 h (SD = 1.3) of MVPA per week and 3.8 h (SD = 1.2) of recreational sedentary behaviour per day. The average sleep duration was 8.7 h per night (SD = 1.1). The mean energy drink consumption score was 1.5 (SD = 1.0), and the mean meal skipping score was 5.2 (SD = 4.0). Among participants reporting past-month cannabis use, 94.4% also reported use of at least one additional substance in the past 30 days.

Compared with non-users, past-month cannabis users reported significantly lower diet quality scores, higher energy drink consumption, greater meal skipping, and higher depressive symptoms and anxiety symptom scores. Cannabis users also reported slightly higher MVPA and longer sleep duration. Full descriptive statistics comparing past-month cannabis users and non-users are presented in Table 1.

### 3.2. Correlations

Bivariate correlations were conducted to examine associations between diet quality and study covariates prior to multivariable modelling. Diet quality was significantly associated with several demographic and behavioural variables, although most correlations were weak in magnitude. The largest associations were observed for meal skipping (r = −0.33, *p* < 0.001), MVPA (r = 0.24, *p* < 0.001), and sedentary behaviour (r = −0.18, *p* < 0.001). Smaller but significant associations were also observed for sleep duration (r = −0.08, *p* = 0.001) and energy drink consumption (r = −0.10, *p* < 0.001). Cannabis use demonstrated a weak negative correlation with diet quality (r = −0.07, *p* = 0.01).

### 3.3. Regression Analysis

Results of the hierarchical multiple linear regression models examining associations between past 30-day cannabis use and diet quality are presented in Table 2.

#### 3.3.1. Model 1

Model 1 examined the association between past-month cannabis use and diet quality after adjustment for demographic characteristics. Cannabis use was associated with lower diet quality (B = −0.81, β = −0.08, *p* < 0.01). Older age was associated with higher diet quality (*p* < 0.001), and males reported significantly lower diet quality compared with females (*p* < 0.001). Among race/ethnicity categories, only participants identifying as Other Ethnicity differed significantly from the White reference group (*p* < 0.001). The model explained 5.4% of the variance in diet quality.

#### 3.3.2. Model 2

Model 2 additionally adjusted for health behaviours, including energy drink consumption, MVPA, sedentary behaviour, and sleep duration. Cannabis use remained significantly associated with lower diet quality, although the magnitude of the association was modestly attenuated (B = −0.70, β = −0.07, *p* = 0.01). Higher energy drink consumption and sedentary behaviour were associated with lower diet quality, whereas higher MVPA was associated with higher diet quality. Sleep duration was not significantly associated with diet quality. The addition of health behaviour variables substantially improved model fit, increasing the adjusted explained variance from 5.4% to 14.6%.

#### 3.3.3. Model 3

Model 3 further adjusted for depressive and anxiety symptoms measured using the PHQ-2 and GAD-2, respectively. Cannabis use remained associated with lower diet quality (B = −0.67, β = −0.06, *p* = 0.01). Depressive and anxiety symptoms were not independently associated with diet quality after adjustment for behavioural variables. Mental health variables contributed minimally to the association between cannabis use and diet quality, with little change in the cannabis coefficient or overall explained variance. The model explained 14.6% of the variance in diet quality.

#### 3.3.4. Model 4

Model 4 additionally adjusted for living arrangement. Cannabis use remained associated with lower diet quality (B = −0.65, β = −0.06, *p* = 0.02). Compared to students living on campus, living with parents was associated with higher diet quality (B = 1.66, β = 0.12, *p* < 0.001), whereas other living arrangements were not significantly associated with diet quality. The addition of the living arrangement variable resulted in a modest increase in explained variance (adjusted R^2^ = 0.154).

### 3.4. Sensitivity Analysis

Sensitivity analyses examining meal skipping and other substance use in associations between cannabis use and diet quality are shown in Table 3.

#### 3.4.1. Model 5

In Model 5, adjustment for meal skipping substantially attenuated the association between cannabis use and diet quality, and the cannabis coefficient was no longer statistically significant (B = −0.44, β = −0.04, *p* = 0.09). Meal skipping was strongly associated with lower diet quality (B = −0.32, β = −0.25, *p* < 0.001) and emerged as one of the strongest behavioural correlates of diet quality in the fully adjusted model. The addition of meal skipping increased the adjusted explained variance from 15.4% to 20.5%.

#### 3.4.2. Model 6

Model 6 additionally adjusted for any other substance use. Cannabis use remained non-significantly associated with diet quality (B = −0.44, β = −0.04, *p* = 0.11). Adjustment for other substance use did not materially alter model estimates or explained variance. The high degree of overlap between cannabis use and other substance use may have limited the ability to isolate independent substance-specific effects. Model 6 explained 20.5% of the variance in diet quality.

## 4. Discussion

This study examined the association between past-month cannabis use and diet quality among university students. The prevalence of past-month cannabis use in the present study (33.7%) is largely consistent with previous research among Canadian university students [4,6]. Cannabis use was initially associated with lower CFIS diet quality scores after adjustment for demographic factors. This association remained statistically significant following additional adjustment for health behaviours, mental health variables, and living arrangements, although the magnitude of the association progressively attenuated across models. However, after adjustment for meal skipping, the association between cannabis use and diet quality was attenuated and no longer statistically significant, and this pattern remained unchanged following adjustment for other substance use. Collectively, these findings indicate that the observed cross-sectional association between cannabis use and diet quality was attenuated after accounting for broader behavioural factors, particularly meal skipping. However, because meal skipping may overlap conceptually with diet quality and may represent a potential pathway between cannabis use and dietary behaviours, attenuation following adjustment should not be interpreted as evidence of mediation or causality. Rather, these findings suggest that irregular eating behaviours represent an important correlate within the broader association between cannabis use and diet quality.

These findings highlight the importance of broader behavioural eating patterns rather than cannabis use alone. Previous research has shown that meal skipping is linked to poorer dietary quality among university students and young adults [2,18] and is associated with lower HEI scores and reduced intake of fruits, vegetables, whole grains, dairy, and protein-rich food groups, indicating poorer overall dietary composition [19]. Consistent with this literature, meal skipping emerged as one of the strongest behavioural correlates of diet quality and substantially attenuated the cannabis coefficient in fully adjusted models. Because meal skipping may plausibly occur along the pathway linking cannabis use with dietary behaviours, adjustment for meal skipping may partially attenuate the association by accounting for variance that could represent an intermediate behavioural process. Therefore, the results from Model 5 should not be interpreted as demonstrating that cannabis use has no relationship with diet quality after accounting for meal skipping, but rather that meal skipping represents an important behavioural factor associated with the observed cannabis–diet quality relationship.

Cannabis use has previously been associated with dietary behaviours, although findings across studies remain inconsistent. Some studies have reported higher intake of energy-dense foods and greater overall caloric consumption among cannabis users [8], whereas others have observed minimal or no differences in diet quality when comparing cannabis users and non-users [20]. These inconsistencies may partly reflect differences in the extent to which studies account for co-occurring behavioural factors. In the present study, the magnitude of the cannabis–diet quality association was reduced after adjustment for behavioural covariates, whereas meal skipping demonstrated a comparatively stronger association with diet quality. This pattern is compatible with a behavioural clustering framework, whereby health-related behaviours such as sleep, physical activity, sedentary behaviour, substance use, and eating patterns may co-occur within individuals rather than occur independently [10]. Cannabis use has also been shown to cluster with other behaviours, including nicotine use, poorer sleep quality, reduced dietary quality, and altered eating routines, all of which may contribute to poorer overall diet quality [8,10,19]. In this context, the present findings support the possibility that eating patterns and broader behavioural routines may contribute importantly to previously observed associations between cannabis use and diet quality.

Living arrangement was also independently associated with diet quality in the present study. Compared with students living on campus, students living with parents reported significantly higher diet quality scores, whereas other living arrangements were not significantly associated with diet quality. This finding is consistent with previous research suggesting that students living at home may have greater access to structured meal routines and home-prepared foods, both of which have been associated with higher dietary quality among young adults [13]. Although living arrangements contributed only modestly to overall model fit, these findings support the importance of contextual factors in shaping dietary behaviours during young adulthood.

This study has several strengths. It included a relatively large and diverse sample of university students, which contributed to stable estimates across hierarchical regression models. The dataset also incorporated a broad range of behavioural, psychological, and contextual variables, allowing for a more comprehensive assessment of diet quality than studies focusing on single exposures. In addition, the hierarchical modelling approach enabled systematic evaluation of how associations changed following sequential adjustment for relevant covariates.

Several limitations should be considered. The cross-sectional design prevents conclusions regarding causality or directionality of associations. Although the study adjusted for a broad range of demographic, behavioural, psychological, and contextual factors, cannabis use was not randomly assigned and may reflect underlying social, behavioural, psychological, or environmental differences that also influence diet quality. Therefore, residual confounding from unmeasured factors, including academic stress, food environment, food access, affordability, and cooking-related resources, may remain. The relatively low survey response rate may also limit generalizability and raise the possibility of selection bias if participating students differed from non-respondents in health-related interests, nutrition awareness, or substance use patterns. Findings should therefore be interpreted as representative of participating students rather than all registered university students.

All measures were self-reported, which may introduce recall and reporting bias. The sample was predominantly female, limiting the ability to examine whether associations between cannabis use and diet quality differ by sex. Although sex was adjusted for in the regression models, future studies with larger and more balanced samples should examine potential effect modification. Similarly, racial/ethnic categories were collapsed due to small sample sizes within several groups, which may have improved statistical stability but may also have obscured meaningful differences between diverse populations. Findings related to race/ethnicity should therefore be interpreted cautiously.

The high prevalence of polysubstance use also limited the ability to isolate cannabis-specific effects. Other substance use was summarized as a binary indicator rather than examined by individual substance categories, limiting the ability to determine whether specific substances contributed differently to observed associations. Additionally, although frequency of cannabis use was collected, past 30-day cannabis use was used as the primary exposure because several frequency categories contained small cell sizes that reduced model stability. This approach is consistent with commonly used indicators of current cannabis use; however, future research should examine whether associations differ according to frequency, quantity, potency, or mode of consumption.

Finally, although living arrangements were included as a contextual variable, more detailed measures of the dietary environment were unavailable. Future studies incorporating measures of food access, affordability, and cooking resources may further clarify factors influencing diet quality among university students. While the fully adjusted models explained approximately one-fifth of the variance in diet quality, this is consistent with behavioural nutrition research demonstrating that dietary patterns among young adults are shaped by multiple interacting social, environmental, behavioural, and psychological factors [2,5,10,11,18,21,22].

These findings have important implications for future research and interventions. Future intervention research targeting meal patterns and broader behavioural routines may represent a promising approach for improving diet quality among university students. Future research should further examine meal timing, eating frequency, and behavioural clustering in relation to diet quality. Longitudinal and experimental studies are needed to clarify the temporal ordering of these relationships and determine whether changes in cannabis use precede changes in dietary behaviours, whether dietary patterns influence subsequent cannabis use, or whether both behaviours are shaped by shared social, environmental, and behavioural factors. Future research examining these pathways may help identify mechanisms underlying associations between cannabis use and diet quality and inform targeted interventions. Overall, this study highlights the importance of considering diet within a broader behavioural and contextual framework among university students.

## 5. Conclusions

Cannabis use was initially associated with lower diet quality among university students; however, this association was attenuated after adjustment for meal skipping and broader behavioural factors. These findings suggest that the observed association between cannabis use and diet quality may reflect broader behavioural patterns associated with dietary habits, although the cross-sectional design prevents conclusions regarding underlying mechanisms or temporal ordering. Meal skipping emerged as a particularly important correlate of lower diet quality, highlighting the importance of eating patterns within behavioural nutrition research. Overall, these findings highlight the importance of considering diet within a broader behavioural and contextual framework among Canadian undergraduate students, while recognizing that findings may not generalize to non-university populations, students in different educational contexts, or youth in other national settings.

## Figures and Tables

**Table 1:** Demographic and Lifestyle Characteristics of University Students by Past-Month Cannabis Use Status (N = 1581).

Variables	Past-Month Cannabis Users (n = 532)	Non-Users (n = 1045)	*p*-Value
**Demographics**			
Age, mean (SD)	21.3 (4.5)	21.7 (5.5)	0.12
Male, n (%)	138 (25.9%)	266 (25.4%)	0.81
White, n (%)	316 (59.4%)	502 (48.0%)	<0.001
South Asian, n (%)	75 (14.1%)	157 (15.0%)	
Chinese, n (%)	38 (7.1%)	154 (14.7%)	
Other ethnicity, n (%)	103 (19.4%)	232 (22.2%)	
**Outcome**			
CFIS score (diet quality), mean (SD)	31.3 (5.0)	32.0 (5.1)	0.01
**Health Behaviours**			
MVPA (hrs/week), mean (SD)	3.0 (1.3)	2.8 (1.3)	0.01
Sedentary behaviour (hrs/day), mean (SD)	3.8 (1.1)	3.8 (1.3)	0.35
Sleep duration (hrs/night), mean (SD)	8.8 (1.1)	8.6 (1.1)	<0.001
Energy drink consumption, mean (SD)	1.8 (1.1)	1.4 (0.9)	<0.001
Meal skipping score, mean (SD)	5.9 (4.0)	4.9 (4.0)	<0.001
**Mental Health**			
PHQ-2 score, mean (SD)	2.1 (1.7)	1.8 (1.6)	<0.001
GAD-2 score, mean (SD)	2.7 (2.0)	2.4 (1.8)	<0.001
**Living Arrangement**			
Alone, n (%)	30 (5.6%)	67 (6.4%)	<0.01
With roommates, n (%)	263 (49.4%)	418 (40.0%)	
With parents, n (%)	67 (12.6%)	191 (18.3%)	
With partner, n (%)	35 (6.6%)	67 (6.4%)	
**Other**			
Other substance use, n (%)	502 (94.4%)	675 (64.6%)	<0.001

Sample sizes vary slightly across variables because of missing responses.

**Table 2:** Hierarchical Multiple Linear Regression Models Examining Associations Between Past 30-Day Cannabis Use and Diet Quality.

Variable	Model 1 (n = 1479)			Model 2 (n = 1475)			Model 3 (n = 1473)			Model 4 (n = 1469)		
	B	β	*p*	B	β	*p*	B	β	*p*	B	β	*p*
**Cannabis use past month**	−0.81	−0.08	<0.01	−0.70	−0.07	0.01	−0.67	−0.06	0.01	−0.65	−0.06	0.02
**Age**	0.16	0.16	<0.001	0.14	0.15	<0.001	0.14	0.14	<0.001	0.12	0.12	<0.001
**Male**	−1.41	−0.12	<0.001	−1.82	−0.15	<0.001	−1.84	−0.16	<0.001	−1.83	−0.16	<0.001
**South Asian**	−0.65	−0.05	0.09	−0.16	−0.01	0.67	−0.14	−0.01	0.70	−0.10	−0.01	0.79
**Chinese**	−0.04	−0.00	0.93	0.44	0.03	0.28	0.44	0.03	0.29	0.66	0.04	0.11
**Other Ethnicity**	−1.19	−0.10	<0.001	−0.69	−0.06	0.03	−0.70	−0.06	0.03	−0.69	−0.06	0.033
**Energy drinks**	-	-	-	−0.47	−0.09	<0.001	−0.46	−0.09	<0.001	−0.43	−0.08	0.001
**MVPA**	-	-	-	0.99	0.25	<0.001	0.97	0.25	<0.001	0.99	0.26	<0.001
**Sedentary behaviour**	-	-	-	−0.52	−0.13	<0.001	−0.50	−0.12	<0.001	−0.50	−0.12	<0.001
**Sleep**	-	-	-	−0.19	−0.04	0.094	−0.19	−0.04	0.10	−0.15	−0.03	0.190
**PHQ-2**	-	-	-	-	-	-	−0.04	−0.01	0.69	−0.03	−0.01	0.72
**GAD-2**	-	-	-	-	-	-	−0.06	−0.02	0.46	−0.07	−0.02	0.41
**Living alone**	-	-	-	-	-	-	-	-	-	0.88	0.04	0.15
**Living roommates**	-	-	-	-	-	-	-	-	-	0.51	0.05	0.10
**Living parents**	-	-	-	-	-	-	-	-	-	1.66	0.12	<0.001
**Living partner**	-	-	-	-	-	-	-	-	-	0.96	0.05	0.16
**Constant**	29.38			30.99			31.21			30.60		
**Adjusted R^2^**	0.054			0.146			0.146			0.154		

Reference categories: female gender, White race/ethnicity, and campus residence.

**Table 3:** Sensitivity Analyses Examining Meal Skipping and Other Substance Use in Associations Between Cannabis Use and Diet Quality.

Variable	Model 5 (n = 1468)			Model 6 (n = 1467)		
	B	β	*p*	B	β	*p*
**Cannabis use past month**	−0.44	−0.04	0.09	−0.44	−0.04	0.11
**Age**	0.10	0.10	0.001	0.10	0.10	0.001
**Male**	−1.83	−0.16	<0.001	−1.83	−0.16	<0.001
**South Asian**	0.25	0.02	0.49	0.24	0.02	0.52
**Chinese**	0.88	0.06	0.03	0.87	0.06	0.03
**Other Ethnicity**	−0.37	−0.03	0.24	−0.38	−0.03	0.24
**Energy drinks**	−0.23	−0.04	0.08	−0.23	−0.04	0.08
**MVPA**	0.85	0.22	<0.001	0.85	0.22	<0.001
**Sedentary behaviour**	−0.38	−0.09	<0.001	−0.38	−0.09	<0.001
**Sleep**	−0.11	−0.02	0.32	−0.11	−0.02	0.32
**PHQ-2**	0.10	0.03	0.28	0.10	0.03	0.29
**GAD-2**	−0.03	−0.01	0.66	−0.03	−0.01	0.66
**Living alone**	0.90	0.04	0.12	0.90	0.04	0.12
**Living roommates**	0.57	0.06	0.06	0.57	0.06	0.06
**Living parents**	1.64	0.38	<0.001	1.64	0.38	<0.001
**Living partner**	0.82	0.04	0.21	0.81	0.04	0.22
**Total meals skipped**	−0.32	−0.25	<0.001	−0.32	−0.25	<0.001
**Any other substance use**	-	-	-	0.04	−0.00	0.91
**Constant**	31.46			31.48		
**Adjusted R^2^**	0.205			0.205		

Reference categories: female gender, White race/ethnicity, and campus residence.

## Data Availability

The data presented in this study are not publicly available due to ethical and confidentiality restrictions.

## Supplementary Materials

The following supporting information can be downloaded at: https://www.mdpi.com/article/10.3390/nu18132210/s1, Table S1: Study Variables and Measurements.

## Abbreviations

The following abbreviations are used in this manuscript:

CFIS	Canadian Food Intake Screener
HEI	Healthy Eating Index
MVPA	Moderate-to-Vigorous Physical Activity
PHQ2	Patient Health Questionnaire-2
GAD2	Generalized Anxiety Disorder-2
