# Supplementary material for: Cannabis Use and Diet Quality Among University Students: The Role of Meal Skipping and Health Behaviours
Source: Nutrients. 2026 Jul 7;18(13):2210. doi: 10.3390/nu18132210 (PMC13364245; doi:10.3390/nu18132210)
Supplement: Supplementary file 1 [file nutrients-18-02210-s001.zip › nutrients-4410333-supplementary.pdf]

Table S1. Study Variables and Measurements

VARIABLES	MEASUREMENTS
Gender	Male (1); Female (2); Self-identify (3); Prefer not to answer (4)
Age	Continuous
Race/ethnicity	White (1); South Asian (2); Chinese (3); Black (4); Filipino (5); Arab (6); Latin American (7); Southeast Asian (8); West Asian (9); Korean (10); Japanese (11); Indigenous (12); Other (13)
Past 30-day alcohol use	0 (1); 1-2 (2); 3-5 (3); 6-9 (4); 10-19 (5); 20-29 (6); 30 (7)
Past 30-day cigarette use	0 (1); 1-2 (2); 3-5 (3); 6-9 (4); 10-19 (5); 20-29 (6); 30 (7)
Past 30-day vape use	0 (1); 1-2 (2); 3-5 (3); 6-9 (4); 10-19 (5); 20-29 (6); 30 (7)
Past 30-day cocaine use	0 (1); 1-2 (2); 3-5 (3); 6-9 (4); 10-19 (5); 20-29 (6); 30 (7)
Past 30-day psychedelic use	0 (1); 1-2 (2); 3-5 (3); 6-9 (4); 10-19 (5); 20-29 (6); 30 (7)
Past 30-day non-prescribed stimulant use	0 (1); 1-2 (2); 3-5 (3); 6-9 (4); 10-19 (5); 20-29 (6); 30 (7)
Past 30-day cannabis use	0 (1); 1-2 (2); 3-5 (3); 6-9 (4); 10-19 (5); 20-29 (6); 30 (7)
Diet quality using CFIS	Canadian Food Intake Screener total score; continuous (0-65); based on frequency of food and beverage group intake over past month
Energy drink consumption past 7 days	Never (1); 1-2 times (2); 3-4 times (3); 5-6 times (4); Once/day (5); 2+ times/day (6)
Meal skipping past 7 days	Breakfast: 0-7 days; Lunch: 0-7 days; Dinner: 0-7 days
Mental health (Patient Health Questionnaire-2)	Continuous score 0-6; higher scores indicate greater depressive symptom severity; based on frequency of “little interest/pleasure in doing things” and “feeling down/depressed/hopeless” over past 2 weeks

Mental health (Generalized Anxiety Disorder-2)	Continuous score 0-6; higher scores indicate greater anxiety severity; based on frequency of “feeling nervous/anxious/on edge” and “not being able to stop/control worrying” over past 2 weeks
Bed time	Before 7pm (1); 7-8pm (2); 8-9pm (3); 9-10pm (4); 10-11pm (5); 11-12am (6); After 12am (7)
Wake time	Before 6am (1); 6-7am (2); 7-8am (3); 8-9am (4); 9-10am (5); After 10am (6)
Hours of moderate/vigorous physical activity per week	0 (1); 1-2 (2); 3-4 (3); 5-6 (4); 7-8 (5); 8+ (6)
Hours of recreational sedentary activity per day	0 (1); 1-2 (2); 3-4 (3); 5-6 (4); 7-8 (5); 8+ (6)
Current living situation	Campus (1); Off-campus alone (2); Off-campus with roommates (3); Off-campus with parents (4); Off-campus with common law/spouse (5)
